# **Outcomes of Autologous Fat Injection Laryngoplasty in Unilateral Vocal Cord Paralysis**

**Published:** 2016-05

**Authors:** Ehsan Khadivi, Mohammad Akbarian, Kamran Khazaeni, Maryam Salehi

**Affiliations:** 1 *Sinus and Surgical Endoscopic Research Center, Mashhad University of Medical Sciences, Mashhad, Iran.*; 2 *Department of Otorhinolaryngology, Mashhad University of Medical Sciences, Mashhad, Iran.*; 3 *Department of Epidemiology, Mashhad University of Medical Sciences, Mashhad, Iran.*

**Keywords:** Autologous Fat, Laryngoplasty, Vocal Cord Paralysis

## Abstract

**Introduction:**

Unilateral vocal cord paralysis (UVCP) is not an uncommon finding. Several procedures are available to manage glottal insufficiency. We conducted a clinical trial to evaluate the outcome of fat injection laryngoplasty.

**Materials and Methods:**

Liposuctioned lower abdomen fat was injected for augmentation of paralyzed vocal cord in 20 patients with UVCP. Autologous fat was harvested with an 18G needle and a 20-ml disposable syringe using a liposuction technique. Clinical follow-up after the injection was carried out from 1 to 6–21 months

**Results:**

Voice and glottal protective function were significantly improved after the surgery. Vocal elements were immediately improved after the surgery, and after 1 year of follow-up.

**Conclusion:**

Fat injection laryngoplasty by liposuction is simple, safe, effective and has a low cost for patients with UVCP with aspiration and breathy voice dysphonia.

## Introduction

Unilateral vocal cord paralysis/palsy (UVCP) is not an uncommon finding in ear, nose and throat (ENT) practice. UVCP is not a diagnosis by itself. The exact incidence of UVCP has been difficult to elucidate for multiple reasons. Many cases are undiagnosed because of spontaneous recovery or compensation by the opposite cord (1).

Idiopathic vocal cord paralysis constitutes the major subgroup of UVCP. Thyroidectomy continues to be the single most common surgical procedure responsible for vocal cord paralysis. Cardiac surgery, trauma, and cerebrovascular accidents also increasingly cause vocal cord paralysis, which is suggestive of the changing trend in lifestyle and life expectancy (2). 

Several procedures are available to manage glottal insufficiency, including vocal fold injection for medialization, medialization thyroplasty, arytenoid adduction, adduction arytenoidopexy, and a variety of reinnervation procedures. Selection of the appropriate procedure depends on the duration of symptoms, severity of impairment, presence of anatomic or surgical defect, and potential for recovery (3). We conducted a clinical trial for the evaluation of short- and long-term outcomes of fat injection laryngoplasty, by serial video laryngoscopy and voice evaluation.

## Materials and Methods

In this study, 20 patients with UVCP from November 2012 to September 2014 were studied for the treatment of autologous fat injection laryngoplasty at the Mashhad University of Medical Science, Mashhad, Iran. Inclusion criteria for UVCP were breathy voice dysphonia and aspiration without structural involvement of the larynx by tumors, lack of vertical displacement of the paralyzed vocal cord, and no contraindication for general anesthesia for any reason. Before surgery, patients were examined by videolaryngoscopy. Voice analysis values were also obtained, including Maximum Phonation Time (MPT), jitter and shimmer.

Under local anesthesia, 50 mL of 2% lidocaine and 1:100000 adrenaline in normal saline solution was injected under the skin of the lower abdomen. Liposuction surgery was performed using an 18-gauge liposuction needle connected to a 20-mL disposable syringe. The resulting liquid was then centrifuged at 2,000 rpm for 4 min. In this way, fat was separated and purified from blood and other substances. 

The patient was then placed under general anesthesia. The upper layer of the centrifuged solution containing solid fat was removed using a 2-mL syringe. Under direct vision and magnification of the larynx with zero-angle laryngeal optic lens, fat was injected into the paraglottic space of the paralyzed vocal cord. The vocal cord was augmented to the midline with a slight overcorrection. Patients were evaluated between 7 days and 1 month later to evaluate improvement in dysphonia and aspiration. Videolaryngo- scopy and voice analysis were repeated 6 to 12 months after treatment. The type and extent of possible side effects of treatment were evaluated during this period. 

All analyses were performed using the Statistical Package for the Social Sciences software, version 20 (SPSS 20).

## Results

All patients had UVCP, breathy voice dysphonia and aspiration of fluids. Eleven patients (55%) had a known cause of vocal cord paralysis. Eleven (55%) cases had left paralyzed vocal cord and nine (45%) patients had right vocal cord paralysis. Thirteen patients (65%) were women and seven (35%) were men. Their ages ranged from 20 to 57 years, with a mean of 43.4 years (Table. 1). 

**Table 1:** Variables

	**Min**	**Max**	**Mean**	**SD**
Age (year)	20	57	43.4	12.0
Injected fat (mL)	1.5	3.0	1.9	0.3
Symptomatic period (month)	5	10	7.1	1.4
Follow-up (month)	6	21	13.6	4.8

Preoperative etiology distribution of the patients is shown in Table 2. The mean volume of injected fat was 1.9 mL (with a minimum of 1.5 mL and a maximum of 3 mL) and the mean follow-up was 13 months (6–21 months). None of the patients had complications from fat harvesting or fat injections at the final examination. Possible improvements in the movement of the vocal cord were noticed. 

**Table 2:** Origins of unilateral vocal cord paralysis among patients

**Origin**	**No. (%) (N=20)**
Iatrogenic	
Thyroidectomy Glomus jugulare tumor Brain tumor Idiopathic	8 (40) 2 (10) 1 (5) 9 (45)

*Resolution of aspiration*

Fluid aspiration was present in all patients, but aspiration of solid foods was observed in 10 patients (50%) before surgery. Thyroplasty using fat injection resulted in a significant improvement in patient aspiration. Six months after surgery, an 80% improvement in aspiration of liquids and 100% improvement in aspiration of solid foods was noticed (P=0.004). Among non-responders (four patients), one patient reported improvement after 4 months. In the improved group (16 patients), one patient reported a return of symptoms after 5 months. These results remained unchanged until the final follow-up.

*Voice quality improvement*

The short- and long-term success of the treatment of dysphonia was equivalent, at 85% (17 patients). One of the improved patients showed some degree of breathy voice dysphonia after 5 months. One patient with no short-term response showed some improvement in aspiration and dysphonia after 4 months. MPT, jitter and shimmer improved significantly (P<0.001) (Table. 3). 

**Table 3:** Postoperative voice quality outcomes

**Variable**	**Baseline** **Mean (SD)**	**1 month follow-up** **Mean (SD)**	**6–12 month follow-up** **Mean (SD)**
Maximum phonation time (s) Jitter (%) Shimmer (dB)	2.7 (1.3) 2.4 (1.4) 6.8 (3.2)	6.4 (2.4) 0.7 (0.7) 3.8 (1.6)	7.6 (1.8)ⁱ 0.5 (0.3) ⁱ 4.0 (1.5) ⁱ

ⁱ Significant different among 1 and 12-month follow-up (P<.001,General Linear Model, Repeated Measure ANOVA test)

*Anatomic medialization*

**Fig 1:**
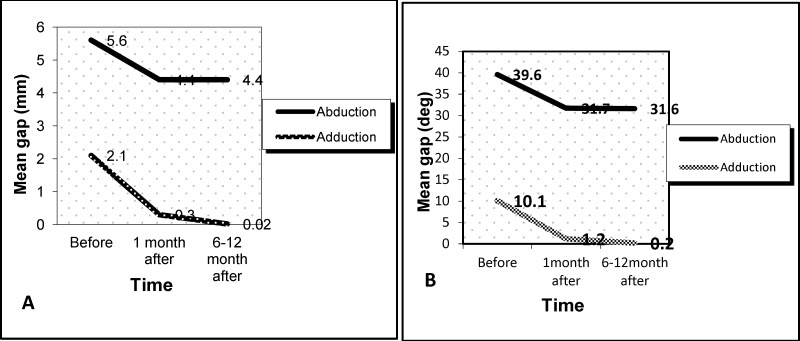
Glottic gap before and after the surgery during adduction and abduction of the vocal cords. (A) Distance in millimeter and (B) angle in degree (P<0.001)

The distance and angle between the vocal cords was measured using video laryngoscopic pictures. To record the distance between the two vocal cords, two-thirds of the front and one-third of the rear of the vocal cords was marked and then measured in millimeters using a ruler. The angle between the vocal cords on the same photo was measured using a conveyor. Postoperative stroboscopic studies showed significant changes in gap and angle between the true vocal cords, as shown in Figure 1 (P<0.001).

## Discussion

 UVCP results from a wide variety of diseases. Surgical trauma, benign and malignant tumors from the skull base to the mediastinum, idiopathic and viral infections are all among the causes of this symptom (4). Patients with UVCP may report breathy voice dysphonia, aspiration, or ineffective cough (3). 

The short- and long-term results of fat injection thyroplasty in this study were satisfactory. Of the 20 patients included, all of whom were suffering from fluid aspiration, 16 (80%) reported improvement 1 month after fat injection. This rate remained the same in the long-term follow-up. In this group, one patient (6.25%) reported that fluid aspiration reappeared after 5 months. In the group of non-responders (four patients), one patient reported recovery in aspiration and dysphonia after 4 months.

The results of solid food aspiration showed a different pattern. Before surgery, 10 patients had solid food aspiration. After 1 month, nine patients (90%) reported improvement while in the long-term (6 months) all 10 patients (100%) showed improvement. Short-term and long-term success of breathy voice dysphonia was 85% (17 patients).

*Voice function*

In voice analysis, patients showed a significant improvement in all parameters. Final examinations were also normal in terms of the appearance of the vocal cords. We noticed improved levels of MPT, jitter and shimmer in both the short- and long-term.

A liposuction method, as reported by Umeno et al, and Sasai et al, was used in this study for the treatment of UVCP with satisfactory results (5,6). 

Fang et al. harvested autologous fat using surgical excision from periumbilical subcutaneous tissue (7). In this way, fat globules were removed from connective tissue and then divided into 1-mm^3 ^particles, which were injected with a high pressure Karl Storz syringe into the vocal cord. Results were reported as favorable.

Sasai et al. reported microscopic examination of the injected fat tissue in the larynx in two patients who underwent total laryngectomy after undergoing the fat injection thyroplasty method (6). The results suggest the viability and sustainability of fat at 12 and 41 months.

Mikus et al. compared the purification method with the liposuction technique of harvesting fat in animal models (8). In the purification method, adipose tissue was surgically removed, and, after washing with normal saline, fat cells of connective tissue were removed. The results of this study indicate that the extracted fat with liposuction has greater longevity. 

In a study by Tucker (9), submental region fat was implanted directly in the larynx, which led to satisfactory results.

Since the absorption of fat has been reported in various studies (6,10,11,12), in the current study, the injections were performed with a slight overcorrection of the vocal cord.

## Conclusion

Treatment of UVCP by autologous fat injection technique improves vocal performance and has a protective role for the larynx. Our study shows that the technique of fat injection laryngoplasty by liposuction is simple, safe, effective, and has a low cost for patients with UVCP with aspiration and breathy voice dysphonia.
